# Chiral nematic liquid crystal droplets as a basis for sensor systems†‡

**DOI:** 10.1039/d1me00189b

**Published:** 2022-03-07

**Authors:** Daniel A. Paterson, Xiaoxue Du, Peng Bao, Adele A. Parry, Sally A. Peyman, Jonathan A. T. Sandoe, Stephen D. Evans, Dan Luo, Richard J. Bushby, J. Cliff Jones, Helen F. Gleeson

**Affiliations:** a School of Physics and Astronomy, University of Leeds Leeds LS2 9JT UK d.a.paterson@leeds.ac.uk h.f.gleeson@leeds.ac.uk; b School of Chemistry, University of Leeds Leeds LS2 9JT UK; c Department of Electrical and Electronic Engineering, Southern University of Science and Technology Shenzhen 518055 China; d Leeds Institute of Medical Research, University of Leeds Leeds LS2 9JT UK

## Abstract

For a series of phospholipid coated calamitic nematic liquid crystal droplets (5CB, 6CB, 7CB, E7 and MLC7023) of diameter ∼18 μm, the addition of chiral dopant leaves the sign of surface anchoring unchanged. Herein we report that for these chiral nematic droplets an analyte induced transition from a Frank–Pryce structure (with planar anchoring) to a nested-cup structure (with perpendicular anchoring) is accompanied by changes in the intensity of reflected light. We propose this system as both a general scheme for understanding director fields in chiral nematic liquid crystal droplets with perpendicular anchoring and as an ideal candidate to be utilised as the basis for developing cheap, single use LC-based sensor devices.

## Abstract 2

For a series of phospholipid coated calamitic nematic liquid crystal droplets the addition of chiral dopant leaves the sign of surface anchoring unchanged but changes the director structure from Frank–Pryce to nested-cup inducing a strong optical change suitable for sensing.

## Graphical abstract

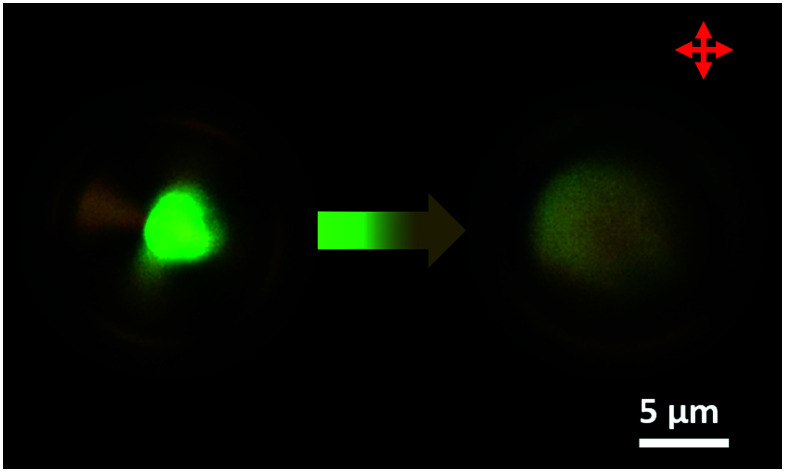

**Design, System, Application**

Point of care testing is becoming an ever more important aspect of modern healthcare. The need for a simple system whereby a diagnosis can be obtained without the need for complicated optical set-ups or read out devices is a crucial step in the design of these inexpensive usable sensor devices. Chiral nematic liquid crystal (LC) droplets are ideal candidates to solve this issue, however this type of system has not yet been utilised for this application. In order to explore this area and aid in the future design of LC based sensor devices we produced a series of monodisperse, surfactant coated, chiral nematic droplets *via* microfluidics. We demonstrate that a change in the surface alignment from planar aligned (Frank–Pryce) to perpendicular aligned (nested-cup) can be chemically induced. This results in a change in the intensity of reflected light. By controlling the LC and surfactant composition, a large degree of control can be extended over the alignment of the LC droplets. The incorporation of a chiral dopant enables the observation of a reflected light intensity change that signals the change in surface anchoring. We propose this as a system by which an LC sensor device can be based.

## Introduction

Most attempts to develop liquid crystal (LC) sensors^1–16^ rely on the fact that the sign of anchoring can be reversed by a very small change at the interface between the LC and surrounding medium, and on the way that this change propagates throughout the whole of a LC thin film, shell^17–19^ or droplet; either as free droplets, trapped droplets^20^ or droplets attached to a surface.^21^ It means that LC-based sensors are potentially very sensitive and naturally amplifying.^6–8^ However, these very attractive advantages need to be set against a number of significant challenges. One issue, specific to the important area of bio-sensing, is that commercial nematic liquid crystals are hydrophobic water-insoluble materials; they are immiscible with biological systems. This can be overcome by coating the interface with a monolayer of a natural or bio-compatible surfactant. Of the available candidates, phospholipids could make it possible to exploit some natural bio-membrane receptors.^22^ LC-based sensor technology also lends itself to point of care testing, meaning development of single-use devices that can be used without expensive polarizing microscopes or flow cytometry apparatus^23^ is viable. LCs can be used to make to very low-cost devices, the outstanding example being LC thermometers.^24^ These exploit droplets of chiral nematic LC dispersed in a polymer matrix and make use of the way in which the selective reflection of visible light (wavelength or colour) depends on temperature. Therefore, an attractive choice for practicable LC sensor systems could be one based on chiral nematic LC droplets that make use of changes in either the wavelength or intensity of the reflected light.^25^ This is the idea which is explored in the present paper using phospholipid-coated chiral nematic LC droplets.

LC droplets show a rich science arising from the interplay between the energetic demands of surface anchoring and the need to minimise the elastic energy within the system. The surface anchoring energy^26^ depends on many factors including both the nature and the density of the surfactant coating. However, this surface anchoring energy is not simply a function of the interaction between the LC molecules and the surface layer. The interaction of LC molecules with each other is also critical; in particular the energy cost arising from the local smectic-like ordering^22,27–29^ imposed by perpendicular anchoring influences the interaction. Whereas the surface energy *F*_s_ scales as ∼ *WR*^2^, where *W* is an anchoring energy coefficient and *R* is the radius of the droplet, the elastic energy *F*_E_ increases linearly with the radius ∼ *KR*. From this a critical radius can be determined *R*_C_ ∼ *K*/*W* above which the surface anchoring energy dominates and below which the elastic energy dominates.^30^ Given typical magnitudes of the mean elastic constant *K* ∼ 10^−11^ N and 10^−5^ N m^−1^ < *W* < 10^−4^ N m^−1^, the critical radius is in the range 1 μm ≤ *R*_*C*_ < 10 μm. In small droplets, where there is strong surface curvature, as well as the normal splay, twist and bend terms *K*_11_, *K*_22_ and *K*_33_, the saddle-splay term *K*_24_ can also be a significant contributor to the overall elastic energy.^31^

**Fig. 1:**
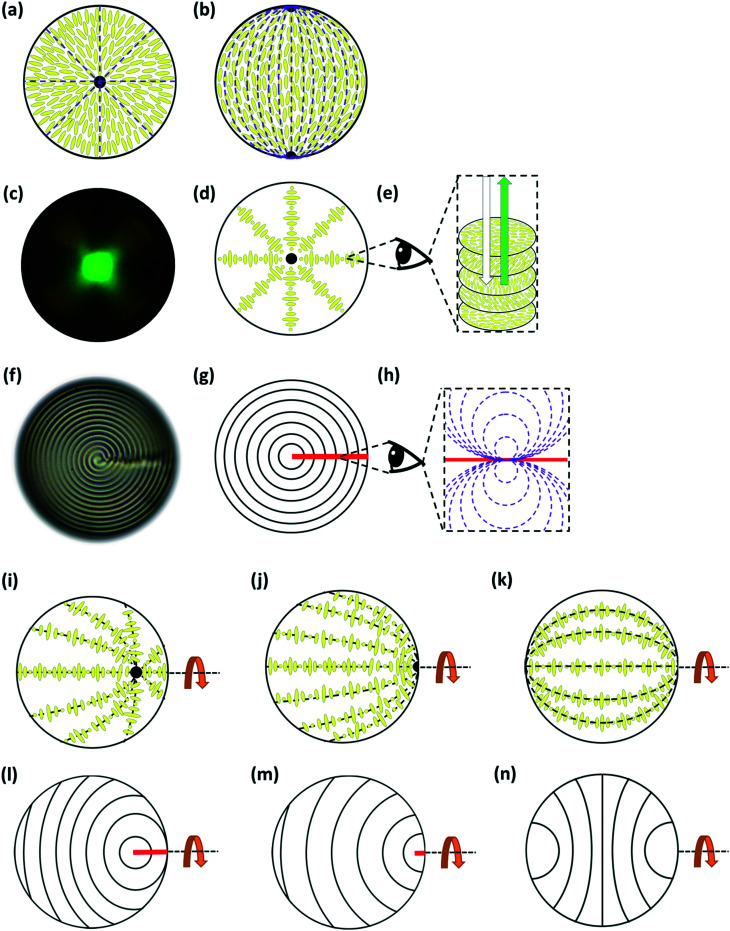
Schematic representations of LC director fields in LC droplets. (a) Nematic LC, radial director field. The LC director is perpendicular to the surface and there is a hedgehog defect at the centre of the droplet. (b) Nematic LC, bipolar director field. The LC director field is planar (parallel) to the interface with two surface defects (boojums) at opposing poles. (c)–(h) Chiral nematic LC droplets with a Frank–Pryce structure. The LC director is planar to the interface but, as shown in (d), the helical axes are radially disposed. (c) Shows a reflection-mode image of a highly chiral Frank–Pryce droplet, where the radius of the droplet accommodates a large number of helical pitches (DOPC : DOPG coated MLC7023 reflecting light at 498 nm). A bright green “bullseye” spot is observed. (e) Layered structure of the director field in chiral nematic LCs showing the twist in the director through *p*_0_/2 (half of the full pitch) (f) shows a transmission-mode image of a Frank–Pryce droplet (DOPC : DOPG coated MLC7023 with *p*_0_/2 = 7.8 μm). Under these conditions, layers of equal twist of the LC director (isocline layers) show up as a series of concentric rings (or as a spiral). These isocline layers are shown schematically in (g). Such structures require an *s* = +2 defect line in the LC director field. This is illustrated in (h). The commonest results of an attempt to impose non-planar anchoring are shown in (i)–(n). (i) and (j) show the dispositions of the helical axes and (l)–(n) show the isocline layers. Unlike the Frank–Pryce droplets the *s* = +2 disclination line in (i) and (l) does not reach the centre of the droplet. In terms of the isocline layers, the structures are symmetrical about the rotational, polar axes shown.

For droplets of (non-chiral) nematic LCs, there are several possible director fields including: radial; bipolar; twisted bipolar; escaped toroidal; toroidal *etc.*^32^ but, the commonest are radial (associated with perpendicular anchoring at the interface) and bipolar (associated with planar anchoring), Fig. 1a and b.

For chiral nematic LC droplets, when there is planar anchoring at the surface, provided the pitch *p*_0_ is less than the radius *R*, a Frank–Pryce structure results Fig. 1c–g. In this the helical axes are perpendicular and the isocline planes (the planes that share the same tilt of the director) are parallel to the surface.^33–35^ Because this has a radial distribution of the helical axes and because of the roughly spherical nature of the isocline layers, it is sometimes also referred to as a radial or spherulitic structure.^36^ When the pitch is in the wavelength range of visible light, these droplets are strongly reflective; the property that is exploited in liquid crystal thermometers. When such droplets are examined using a microscope operating in reflection-mode each droplet shows a bright spot (a ‘bullseye’) in its centre, Fig. 1c. This corresponds to the relatively small range of incidence/reflection angles where the helical axis is parallel (or almost parallel) to the viewing axis. Because it is an approximately spherically symmetric structure, the observed reflectance is not affected by tumbling motions of droplets in solution. In cases where the pitch is of the order of a few microns, when the droplets are viewed using transmission-mode microscopy the isocline layers can be visualised as a series of approximately concentric shells, Fig. 1f.

When surfactants are added that change the surface anchoring to (or towards) the perpendicular; more than one structure can result and the literature is not clear on how these relate to each other or why sometimes one structure is formed or another. Some of the most commonly observed director fields are shown in Fig. 1i–n.^37–41^ If the pitch is of the order of the wavelength of visible light and these droplets are observed in reflection-mode, most often what is seen is the so-called ‘flashlight’ structure; a single reflecting spot, which appears to be located on the surface of the droplet but which moves as the droplet tumbles in solution and periodically appears and disappears. These droplets are associated with ‘nested-cup’ director fields of the type shown in Fig. 1i, j, l, and m.^37,39,41,42^ Sometimes, however, so-called equatorial structures are observed Fig. 1k and m.^37,39–42^ As well as the nested cup and equatorial structures shown in Fig. 1, in which the focus of the helical axes is inside or at the edge of the droplet, examples are also seen in which it lies outside the droplet. The sequence 1i to 1j to 1k involves increasing alignment of the helical axes with the curved surface of the droplet.^39^ Where this process stops and which structure is finally obtained depends on the strength of the anchoring and the values of all three of the elastic constants; *K*_11_, *K*_22_, and *K*_33_. All represent states in which the helical axes align or begin to align with the curved surface of the droplet;^43^ states in which the surface alignment becomes increasingly biased towards the perpendicular.

**Fig. 2:**
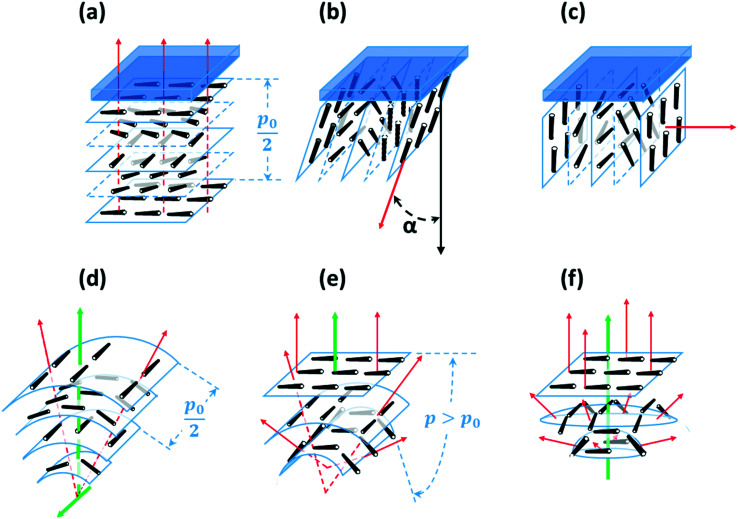
Chiral nematic isocline orientation in Cartesian (a–c), cylindrical (d and e) and spherical (f) systems. (a)–(c) Planar interface. Effect of isocline tilt, showing (a) *α* = 0°, (b) 0° < *α* < 90° and (c) *α* = 90°. (d)–(f) The effect of isocline curvature: (d) coaxial cylindrical layers with the same focal line and therefore constant pitch *p*_0_. (e) Coaxial cylindrical isoclines with a focal line that varies position for each isocline along the axis, (f) coaxial spherical layers in which the focal point varies for each isocline along the axis, also leading to an increased pitch. The co-axis and focal line are shown in green, and the local orientations of the helical axes (isocline normals) are shown as red dashed lines.

If the helical axis of a chiral nematic LC (*i.e.* the isocline normal) is at an angle *α* to a surface normal, the LC directors make an angle to the surface that lies in the range 0° to (90 − *α*)°, so that, if the helical axis is perpendicular to the surface (the isocline planes parallel to the surface) there is planar anchoring of *n* but, if the helical axis is parallel to the surface (the isocline planes perpendicular) the LC *n* directors are evenly distributed between 0° and 90° to the surface normal, Fig. 2.

**Fig. 3:**
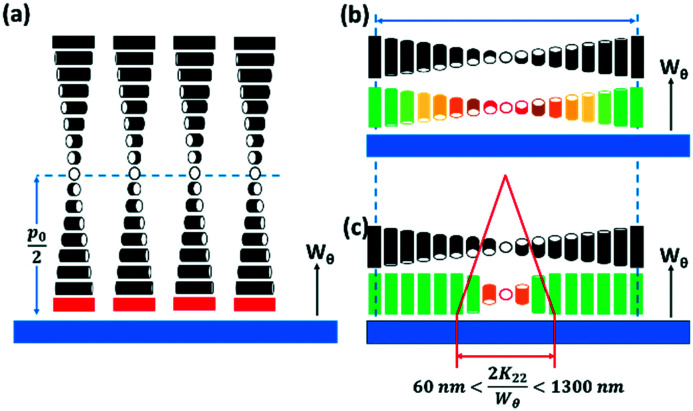
The effect of homeotropic anchoring on chiral nematic liquid crystal alignment. (a) Helical axes perpendicular, isocline layers parallel to the surface. High surface energy cost since the director at the surface is normal to the preferred alignment direction. This is represented by the red colour of the surface director. (b) Helical axes perpendicular, isocline layers parallel to the surface. The surface director alternates between lying parallel (indicated by the green colouration) and perpendicular (indicated by red) to the preferred alignment direction twice per pitch. Between these orientations the director is tilted at an intermediate angle to the surface normal, and the overall energetic cost is half that of the first case. (c) If the anchoring energy is sufficiently high, the helical winding is concentrated towards the high surface energy region, with a distortion width given by the anchoring extrapolation length 2*K*_22_/*W*_*θ*_.

For a surface with perpendicular anchoring and with an anchoring energy *W*_*θ*_, the surface energy density *U*_S_ is of the form;1
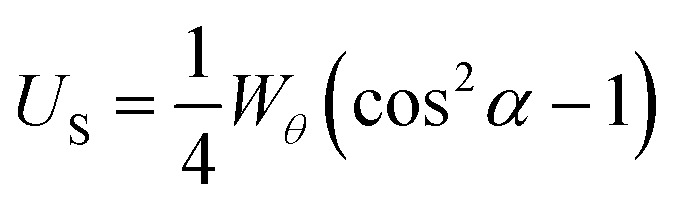
which gives *U*_S_*=* 0 for *α* = 0°, and *U*_S_*=* −1/4*W*_*θ*_ for *α* = 90°. This is the minimum value unless there is distortion of the helix, as may occur for very strong perpendicular anchoring. The helical winding is concentrated towards the high surface energy region, with a distortion width given by the anchoring extrapolation length 2*K*_22_/*W*_*θ*_, Fig. 3c. For reasonable estimates of *K*_22_ and *W*_*θ*_ this leads to a value between 100 and 1000 nm. For curved surfaces, the same local conditions apply, but with additional factors associated with the radius of curvature for the surface and of the isoclines. For the cylindrically curved chiral nematic shown in Fig. 2d, where the isoclines have different radii, *r* but radiate from a single focal line (indicated by the point of intersection for the helical axes shown in red) the pitch remains constant. The isoclines with the director oriented perpendicular to the focal line have an elastic energy contribution from bend *K*_33_ that is proportional to 1/*r*, whereas the isoclines for which the director is parallel to the focal line have no bend contribution. Fig. 2e shows what happens when coaxial cylindrical isoclines vary their curvature to give continually varying focal points on the common axis. Here, the pitch remains the same only along the axis, and diverges in the direction parallel to the plane of curvature. This is also true in the case of coaxial spherical isoclines, shown in Fig. 2f, wherein the increase of pitch occurs in all directions from the axis. It is clear from the structures of Fig. 2e and f that changing the pitch by changing the isocline curvature not only has a cost in twist energy related to *K*_22_, but also splay *K*_11_ for isoclines with the director in the plane of the curvature. For spherical curvature Fig. 2f there is a bend contribution related to *K*_33_/*r* for all isoclines. The twist energetic cost increases with distance from the axis, whereas the splay energy is constant and related to the change in curvature and *K*_11_/*r*.

This paper is concerned with switching between Frank–Pryce and ‘nested-cup/flashlight’ structures caused by changing the surface anchoring from planar to perpendicular. In the Frank–Pryce structure the defect is locked into the centre of the droplet by the surface anchoring. However, once this constraint is relaxed by weakening or changing the sign of the anchoring it is free to migrate towards, to or even beyond the edge of the droplet. Even if the helical axes remain straight,^37^ this will stabilise the system because the total energy within the droplet is reduced (the proportion of highly curved isocline layers is reduced) and the isoclines now intercept the droplet surface (*α* ≠ 90°) which decreases the anchoring cost (eqn (1)). Straight helical axes imply that all of the isocline shells are equally spaced and all centred on the same focus as the helical axes. However, examination of transmission mode images for droplets with a long enough pitch to be visualised,^40–42^ shows that this is not the case. The focus of the second isocline shell is further out from the centre than the first, the third further still and so on. This corresponds to a flattening of the isocline layers, Fig. 2f, a progression away from strained curved isocline layers towards an unstrained state in which they are flat. Clearly this must be true for the ‘equatorial’ structure, Fig. 1k, and if it is also true for the nested cup structures then the trend from Fig. 1d to Fig. 1i to Fig. 1j to Fig. 1k corresponds to a sequence in which the helical axes are increasingly bent and increasingly align with the curved surface of the droplet. A consequence of this is that the isocline layers are no longer evenly spaced; the pitch is stretched and/or compressed. Certainly there should be a change in the spread of wavelengths of the reflected light and perhaps an overall red or blue shift.

**Fig. 4:**
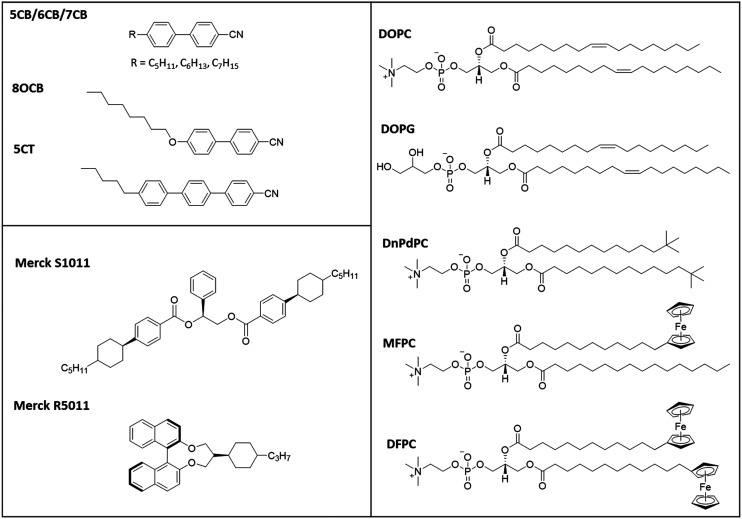
Chemical structures of the LCs, chiral dopant and lipids used (excluding the commercial mixture MLC7023). The composition of E7 is 51% 5CB, 25% 7CB, 16% 8OCB and 8% 5CT.^20^ The composition of MLC7023 is not published but its physical properties have been previously studied and reported.^22^

In this study we have used cyanobiphenyl LCs (5CB, 6CB, 7CB, E7 and MLC7023)^22^ doped with S1011 or R5011, Fig. 4. The phospholipid coatings used were both natural (DOPC and DOPG) lipids and synthetic (DnPdPC, MFPC and DFPC)^22^ lipids shown in Fig. 4.

Since the focus of our work is droplets that are potentially relevant to biosensing applications we have concentrated on monodisperse phospholipid-coated droplets generated in a microfluidic device. However, we have also made use of the fact that strong planar anchoring can also be achieved by using polyvinyl alcohol (PVA) and strong perpendicular anchoring by using sodium dodecyl sulfate (SDS). In the first part of this paper, we establish that, the anchoring for the chiral-doped nematic LC droplets can be manipulated in exactly the same way as that for non-chiral nematic LC droplets.^22^ We then go on to study the effect of switching the sign of the anchoring on the optical properties of the droplets showing that, in the switch from Frank–Pryce to ‘flashlight’ droplets, there may be a shift in the wavelength of the reflected light but that the decrease in intensity is a much more marked and potentially the more useful phenomenon.

## Experimental procedures

### Materials

The LCs 4-cyano-4′-pentylbiphenyl (5CB), 4-cyano-4′-hexylbiphenyl (6CB), 4-cyano-4′-heptylbiphenyl (7CB) and the mixture E7 were purchased from Synthon Chemicals GmbH & Co. KG, Germany. The LC mixture MLC7023 and the chiral additives S1011 (helical twisting power, HTP, 34 ± 2 μm^−1^)^44^ and R5011 (HTP, 120 ± 10 μm^−1^)^45^ were provided by Merck Group, UK.

1,2-Dioleoyl-*sn-glycero*-3-phosphocholine (DOPC) and 1,2-dioleoyl-*sn-glycero*-3-phospho-*rac*-(1-glycerol) sodium salt (DOPG), Texas Red-DHPE and HEPES (purity >99.5%) were purchased from Sigma-Aldrich. The lipids 1,2-di-*neo*-pentadecoyl-*sn-glycero*-3-phosphocholine (DnPdPC), 1-palmitoyl-2-(12′-ferrocenyl)-dodecoyl-*sn-glycero*-3-phosphocholine (MFPC), and 1,2-bis(12′-ferrocenyl)-dodecanoyl-*sn-glycero*-3-phosphocholine (DFPC) were prepared previously.^22^

Premium glass microscope slides and glycerol, were obtained from Fisher Scientific (Pittsburgh, PA). Sylgard 184 silicone elastomer was purchased from Farnell, UK. Polyvinyl alcohol (PVA) (*M*_W_ 30–70 kg mol^−1^, hydrolysis 87–90%), sodium dodecyl sulfate (SDS) and dichloromethane (DCM) were purchased from Sigma Aldrich. All aqueous solutions were prepared using deionized water from a Milli-Q water purification system (Millipore, Bedford, MA).

### Preparation of lipid liposomes

To make the phospholipid-coated LC droplets, a liposome solution was made by hydration and tip sonication of a dried lipid or mixture of lipids with 0.1 mol% Texas Red–DHPE, as described previously.^34^ The Texas Red-DHPE is included to allow fluorescence imaging of the lipid-coated droplets, thereby allowing confirmation of the presence (or absence) of a lipid monolayer coating to the droplet. The lipid mixture was dissolved in a chloroform/methanol 1 : 1 mixture in a glass vial and dried under nitrogen flow for 1 h. Milli-Q H_2_O was then added, and the vial was vortex-mixed for 2 min to produce a suspension of the lipids. The suspension was tip-sonicated for 30 min at 4 °C until a clear solution could be observed and then centrifuged for 5 min at 5000 rpm. The resultant supernatant was decanted to remove metal sediment from the tip sonicator. The supernatant was diluted with H_2_O to give a concentration of lipid of 0.5 mg mL^−1^. 15% volume of glycerol was added to the liposome solution to increase the viscosity, optimizing the flow properties for microfluidic liquid crystal droplet production.

### Preparation of LC chiral mixtures

The mixtures listed in Table ST4 of the ESI† were prepared by co-dissolving weighed amounts of each component in DCM and allowing the solvent to evaporate until dry. The mixtures were further dried under vacuum for at least 24 h prior to characterisation. Mixtures were chose to give either low-pitch droplets (*p*_0_/2 ≈ 7.8 μm) in which the layered structure could be visualised or high-pitch droplets reflecting green/yellow light (*p*_0_ ∼ 350 nm).

### Microfluidic device fabrication

The detail of the fabrication of the polydimethylsiloxane (PDMS) devices used can be found in our previous paper.^20^ Briefly, the software “CleWin” was used for the design. An MW2 laser direct-write laser system (Durham Magneto Optics Ltd, Durham, UK) was used to pattern the SU8 (thickness of 25 μm) creating an SU8 on silicon master. A PDMS (PDMS monomer and curing agent used in a weight ratio of 10 : 1) copy of this silicon master was cross-linked at 75 °C for 1 h. Afterwards, access holes were punched into the PDMS copy, which was then given O_2_ plasma treatment (100 W, O_2_ pressure 0.5 mbar, 1 min, Zepto Plasma Unit, Diener Electronic, Germany) and bonded onto a glass slide. Post-bonding baking in an oven at 75 °C for 30 min increased the bonding strength and resulted in a device ready for use. For the flow focus design used in our experiments, the width of nozzle is about 15 μm and the width and length of channel after the nozzle are 100 and 4000 μm, respectively. The depth of all the channels is 25 μm.

### Thermal characterisation of the liquid crystals

The phase behaviour of the LC chiral mixtures was studied by differential scanning calorimetry (DSC) using a TA Instruments Q20 differential scanning calorimeter equipped with an AQ20 autosampler and a RCS90 refrigerated cooling system, calibrated by use of indium standards. The thermograms were obtained during heating and cooling scans at 10 °C min^−1^ with a 3 min isotherm between heating and cooling segments, under a nitrogen atmosphere. All samples were measured in duplicate. Transition temperatures and associated enthalpy changes were extracted from the third heat trace, and those listed in the ESI† are an average for both samples measured.

### Liquid crystal droplet production

**Fig. 5:**
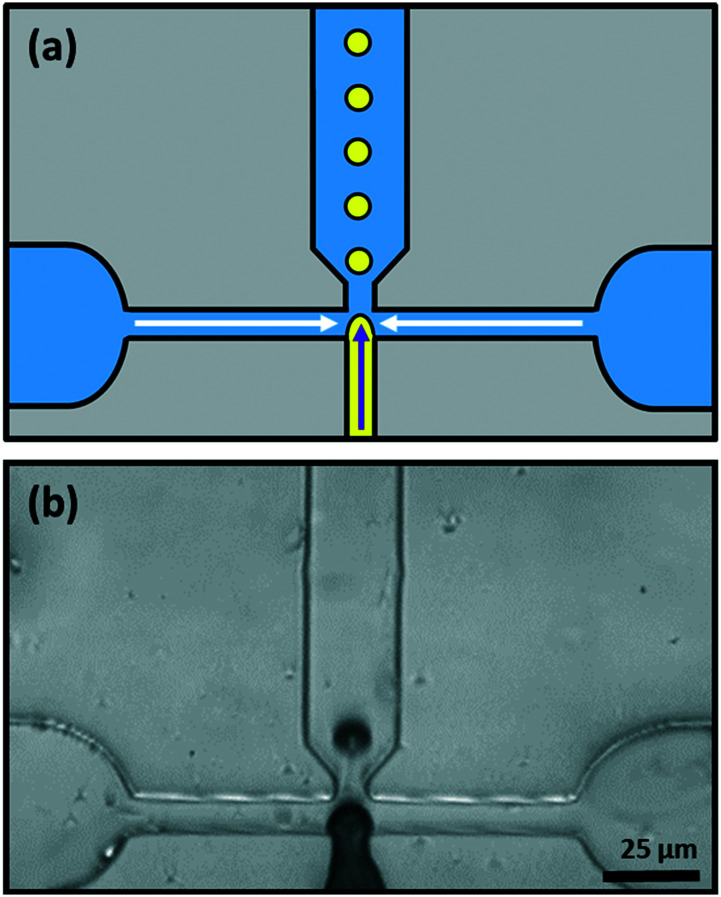
The microfluidic system used for producing LC droplets. (a) Schematic of the device (LC, yellow; aqueous solutions, blue). (b) Microscope image of droplet formation in the microfluidic device, taken using a high-speed camera.

Monodisperse lipid-coated droplets (diameter either = 17 μm or 24 μm) were produced using a flow focus droplet microfluidic device.^46^ A schematic diagram of the droplet formation process in the device is shown in Fig. 5a. The droplet formation device had two inlets. One inlet supplied the two outer side channels, with buffer solution containing lipid in the form of small unilamellar liposomes. The middle inlet was used for the introduction of the LC. The LC and liposome solutions were pumped into the device through the two inlets using two PHD ULTRA advanced syringe pumps (Harvard Apparatus, USA). The flow rate used for LC droplet generation was 0.075 μL min^−1^ for LC and 10 μL min^−1^ for buffer with liposomes. The LC material entered the nozzle and was pinched-off by the buffer solution surrounding it due to shear force.^35^ Lipid vesicles in solution adsorbed and ruptured at the hydrophobic LC/water interface to form a monolayer on the surface, Fig. 5a and b.

Polydisperse LC droplets were formed by using mechanical methods, as reported elsewhere,^47^ usually by deploying a combination of vortexing and agitation by hand. 2 μL of LC in 100 μL of continuous aqueous solution of lipids was enough to lead to a sufficiently dilute emulsion of LC droplets with minimal coalescence and a variety of droplet sizes.

### Optical studies of surfactant-laden liquid crystal droplets

The LC droplets were suspended in aqueous solution and held between a glass microscope slide and a glass coverslip separated by a ∼400 μm deep polydimethylsiloxane (PDMS) well. To make the well, a PDMS thin film was formed by pouring 2 g of fully mixed elastomer and curing agent (weight ratio 10 : 1) (Dow Corning Sylgard 184 kit) into a plastic Petri dish (100 mm in diameter) and leaving at room temperature for 48 h on a horizontal surface. Then, a piece of PDMS thin film (1.5 × 1.5 cm^2^) was cut from the PDMS film in the Petri dish. A hole of 9 mm in diameter was made using a homemade punch. The PDMS sheet was placed on the microscope slide. The LC droplet sample was deposited into the middle of the hole using a pipette, and the top was sealed with a coverslip (0.12 mm thick). Droplets were initially heated into the isotropic phase and then cooled to room temperature (25 °C) at 1 °C min^−1^. All subsequent studies were performed at room temperature except where stated. Optical characterization of the droplets was performed using a DM 2700 M (Leica Microsystems Ltd.) polarized light microscope equipped with a pair of linear polarizers and a Nikon D3000 camera, combined with a Linkam T95 Peltier hot stage. The fluorescence signal from the Texas Red-DHPE fluorophores included in the lipid layers was observed by using an epifluorescence microscope (Nikon Instruments Europe B. V., Kingston, UK) equipped with a Texas Red filter block. Fluorescence images were captured by using an Andor Zyla sCMOS camera (Oxford Instruments plc, UK).

## Results and discussion

### Preparation of monodisperse chiral-doped LC droplets

**Fig. 6:**
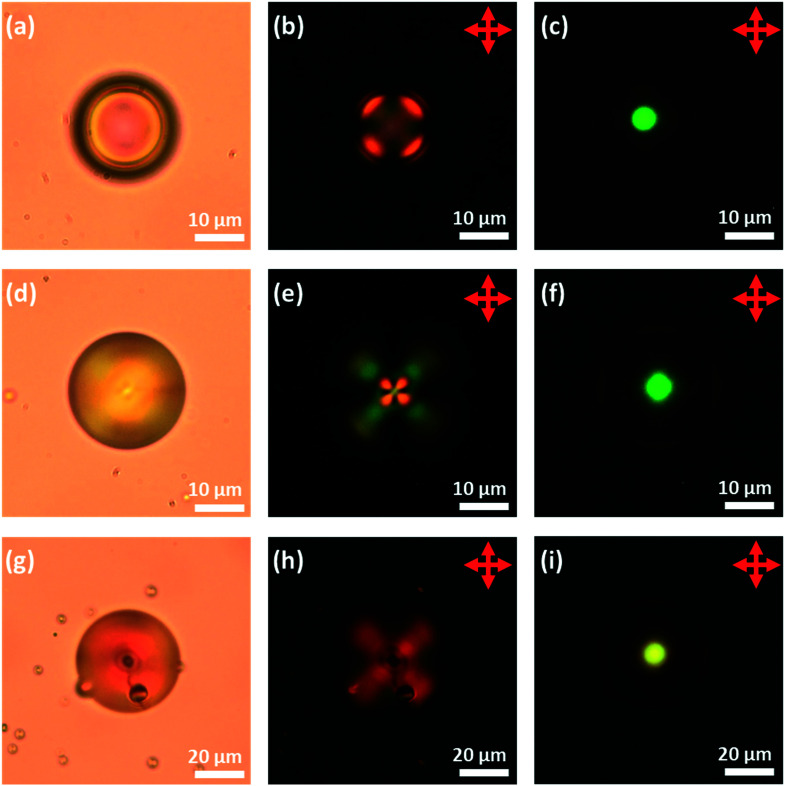
The images of PVA coated LC droplets doped with S1011. (a), (d) and (g) ‘bright field, transmission-mode, (b), (e) and (h) transmission-mode, between crossed polarizers and (c), (f) and (i) reflection-mode, between crossed polarizers. (Top row) 5CB, (middle row) 6CB and (bottom row) 7CB. These droplets were produced using the “shaker method” resulting in a polydisperse sample.

Although we have made some use of LC droplets that were obtained by simply shaking a mixture of a LC and an aqueous solution of the surfactant (Fig. 6 and 11), most of the LC droplets were produced using a PDMS microfluidic device with a flow-focus configuration, Fig. 5.^20,46^ A schematic diagram of this device is shown in Fig. 5a and an image of the device in use is shown in Fig. 5b. The middle inlet channel was used to feed the LC to the pinch-off point where it meets the flow of the two channels either side that feed in aqueous solutions of PVA or of phospholipid liposomes (solution of liposomes ∼25–30 nm in diameter).^48^ Down-stream, the droplets formed at the nozzle become coated with PVA or with a lipid monolayer. This stabilizes the droplets and prevents them from coalescing with each other.

There are several advantages in producing LC droplets using microfluidics, rather than by simply shaking or agitating a mixture of a LC and an aqueous solution of the surfactant. Firstly, the size of the droplets is easy to control by managing the flow-rates.^20^ Secondly, and more importantly, it produces droplets that are uniform in terms of their sizes. This monodisperse nature of the droplets is important in the present case because the director fields in chiral nematic droplets can be dependant on the ratio of the diameter to the chiral pitch. Also, as shown below, producing monodisperse droplets makes it possible to produce well-ordered colloidal crystal arrays. Furthermore, when droplets are produced by agitating a mixture of the LC and a solution of the surfactant, this not only produces an emulsion of LC in water but also an emulsion of water in LC; the LC droplets contain inclusions of small droplets of water that can affect their properties.^22,49,50^

### Control of the director field in chiral-doped LC droplets

First, to obtain droplets with strong planar anchoring and a well-defined Frank–Pryce structure, a PVA coating was employed. The liquid crystals 5CB, 6CB, 7CB, E7 and S1011 were doped with sufficient S1011 to obtain a pitch giving a selective reflection in the yellow/green visible region of the spectrum. This could be done without significantly depressing the clearing point; the phase transitions of the LCs were found to be depressed by about 0.64 °C/wt%, corresponding to about 5 °C. It was also shown that, for these mixtures, between room temperature and the transition temperature, neither the wavelength of the reflected light nor the refractive indices of the LC changed significantly; the mean refractive index only changes by 0.01 across the whole temperature range. Room temperature, optical microscope images taken of isolated droplets of 5CB, 6CB and 7CB doped with S1011 and coated with PVA are shown in Fig. 6. These confirm that there is planar anchoring at the surface of the LC and that the droplets adopt the (expected) Frank–Pryce structure. In some of the bright-field transmission-mode images obtained, the *s* = 2 disclination line is clearly visible, Fig. 6g. When viewed in transmission between crossed polarizers, these droplets looked somewhat similar to non-chiral nematic droplets which have a radial director field, Fig. 6b, e and h. This is because the chiral nematic acts as a negative uniaxial material with the optic axis parallel to the helical axis, thereby giving a simple Maltese cross pattern when aligned parallel to the transmitted light. However, these chiral droplets look very different to non-chiral droplets when they are observed in reflection-mode. Each of them shows a single bright coloured reflected spot (a bullseye) in its centre, Fig. 6c, f and i.

**Fig. 7:**
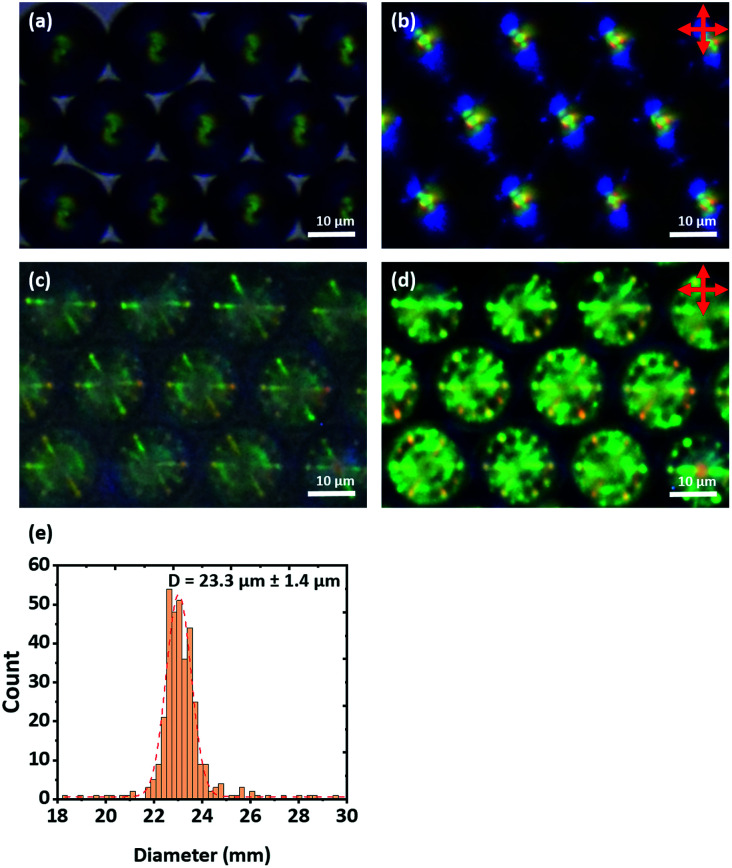
A monolayer of PVA coated E7 droplets doped with S1011 produced microfluidically. (a) and (b), transmission-mode images without and with crossed polarizers. (c) and (d), reflection-mode without and with crossed polarizers. (e) Analysis of the droplet size distribution. The red line is a Gaussian fit to the data. PDI = 0.083.

**Fig. 8:**
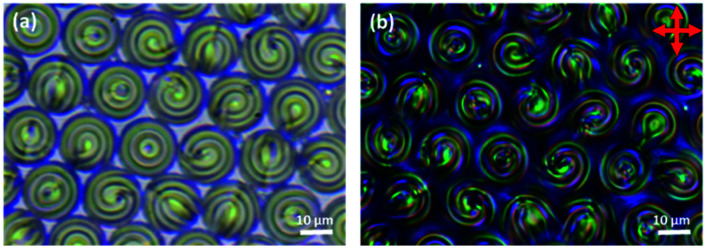
A monolayer of PVA coated 5CB droplets doped with 0.53 wt% of S1011 produced microfluidically. (a) Bright field transmission-mode image and (b) transmission-mode image with crossed polarizers.

Since the droplets that were prepared using microfluidics were monodisperse, it was found that, for a high enough concentration, they tended to form well-ordered hexagonal monolayers, Fig. 7 and 8. When viewed in reflection-mode, although the isolated droplets of E7 doped with S1011 showed a single green bullseye in their centre, the optical properties of this hexagonal array of droplets are much more complex. This is because of photonic cross-communication between close-neighbour droplets. The way in which the various colours and patterns shown in Fig. 7c and d arise has been discussed in detail by Noh *et al.*^51^ Similar hexagonal arrays could also be produced for droplets of 5CB, 6CB and 7CB doped with of S1011.

When the level of the chiral dopant is reduced approximately ten-fold (to obtain droplets in which the isoclines become visible) the presence of the dopant has almost no effect on the clearing temperature of the LC. Because of the much longer pitch, the repeating chiral structure within the Frank–Pryce structure can be resolved and easily seen using bright-field transmission-mode microscopy, Fig. 8. On heating the droplet solution, the Frank–Pryce structure becomes disordered and eventually there is a transition into the isotropic phase. On cooling back from isotropic phase, the well-ordered Frank–Pryce structure re-appears. The monodispersity of the droplets obtained for the sample shown in Fig. 8 was good (diameters 17.7 ± 0.5 μm) and, once again, hexagonal close-packed monolayers could be obtained, Fig. 8. However, because of the much longer pitch, there is no visible photonic cross-communication between droplets. The isoclines are resolved and can be clearly seen in Fig. 8. For some of the droplets (depending on their orientation) the *s* = 2 disclination line is also clearly visible.

**Fig. 9:**
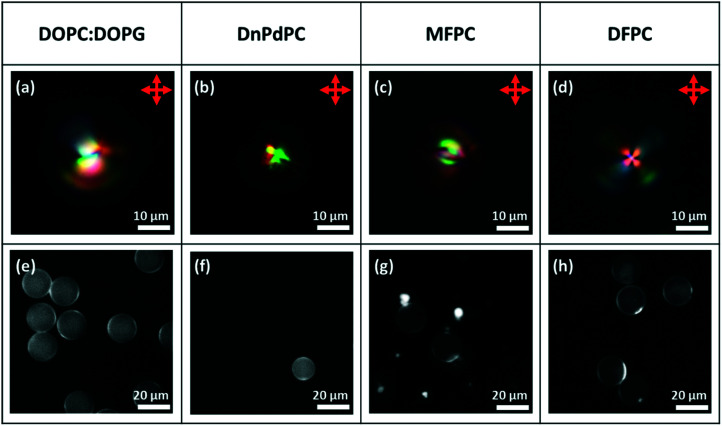
Microfluidically produced 5CB droplets doped with ∼7.3 wt% of S1011 and coated with the lipid surfactant type detailed in the top row. (a)–(d) Transmission-mode images between crossed polarizers; (e)–(h) fluorescence images.^22^

**Fig. 10:**
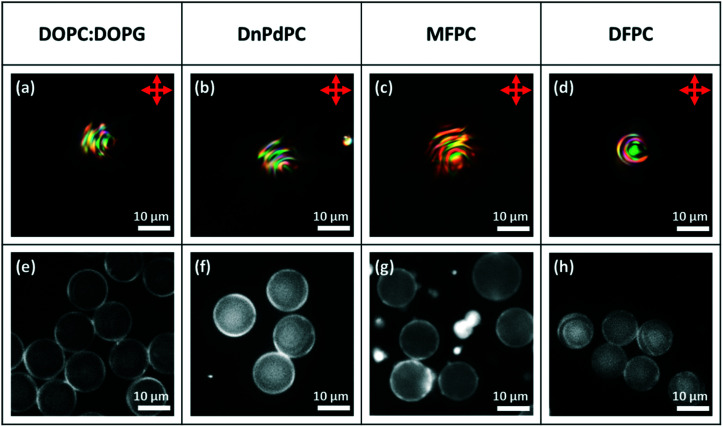
Microfluidically produced 5CB droplets doped with 0.53 wt% of S1011 and coated with the lipid surfactant type detailed in the top row. (a)–(d) Transmission-mode images between crossed polarizers; (e)–(h) fluorescence images showing a clear bright ring around the droplets which can be assigned to a lipid monolayer.

These experiments using a PVA-coating, established that it is possible to prepare monodisperse Frank–Pryce droplets and to prepare well-ordered monolayers. However, PVA-coated droplets are unlikely to be useful in sensor applications and so equivalent droplets with phospholipid coatings were also investigated. Our previous work had established that director fields in non-chiral nematic LC droplets could be controlled either through choice of the LC or through choice of the phospholipid.^22^ Hence, when 5CB, 6CB and 7CB coated with 1 : 1 DOPC : DOPG or DnPdPC or MFPC perpendicular anchoring is observed (there is a radial director field, Fig. 1a) but when the coating is DFPC, it is planar (there is a bipolar director field, Fig. 1b). DFPC has two bulky terminal ferrocenyl residues at the ends of the acyl chains, which are expected to make interdigitation of the LC into the lipid chains more difficult. Perhaps for this reason, the usual preference for perpendicular anchoring is suppressed. Planar anchoring can also be achieved by changing the choice of LC to MLC7023 coated with DOPC : DOPG. It has been shown for non-chiral LCs, that, for this system, anchoring is planar (there is a bipolar director field, Fig. 1b). This ability to reverse the normal anchoring of LCs on phospholipid monolayers is important for potential sensor applications. Potentially, a major problem in using phospholipid-coated droplets is that, for most LC/lipid combinations perpendicular anchoring is very strong and a very strong preference for this mode of anchoring creates difficulties in switching the anchoring to planar. Hence, it is important to demonstrate that, as in the non-chiral droplets, planar anchoring can also be achieved for these phospholipid-coated droplets. This was found to be the case and crucially anchoring in the chiral-doped LC droplets could be controlled in exactly the same way as in the case of their non-chiral counterparts. Fig. 9 shows isolated droplets of 5CB doped with ∼7.3 wt% of S1011 and the effect of changing the phospholipid monolayer (DOPC : DOPG, DnPdPC MFPC or DFPC). In each case, to help establish that a lipid monolayer had formed, a small percentage of Texas Red-DHPE was added to the lipid. The fluorescence microscopy images of the droplets, Fig. 9e–h, show a fairly uniform bright ring around each droplet, confirming that this is indeed the case. In agreement with the previous findings for non-chiral nematic LC droplets, only the droplets coated with DFPC, showed planar anchoring and a Frank–Pryce director field Fig. 9a, and only in this case was a simple Maltese cross observed using transmission-mode microscopy between crossed polarizers. The other three lipids all promote strong perpendicular anchoring at the surface disfavouring a Frank–Pryce director field. In these cases, as expected, the observed textures are more complex, Fig. 9b–d. In the case of the MFPC droplets we found that the samples were ‘contaminated’ with very small highly fluorescent droplets; presumably excess liposomes that could not be removed. In the case of 5CB doped with 0.53 wt% S1011 a similar dependence of anchoring on the choice of LC was observed but, because the pitch is longer than the wavelength of the light, the distinction between Frank–Pryce and non-Frank–Pryce structures can be seen more directly, Fig. 10. Only the DFPC-coated droplets show a Frank–Pryce director field, Fig. 10d. The lipids MFPC, DnPdPC and DOPC : DOPG all promote perpendicular anchoring, giving a ‘nested-cup’ structure. With surfactants such as phospholipids it is known that the strength of anchoring and the stability of the droplets is dependant on the concentration of surfactant in the surrounding medium.^2^ If the concentration is too low, variable anchoring results are obtained and the droplets are unstable; they fuse with each other and with the walls of the container. Based on our previous work we have standardised on phospholipid concentrations that avoid these problems. In cases where the phospholipid concentration is too low we find that the ring observed in flurorescence studies is non-uniform. However, all four of the lipids give a uniform coating in this case, as shown in the fluorescence microscopy images, Fig. 10e–h, but, as before there were issues for the MFPC droplets where we found that the samples were ‘contaminated’ with very small highly fluorescent droplets that could not be removed.

Hence, using these 5CB-based droplets, it is possible to control the director field using custom designed lipids in exactly the same way as for their non-chiral counterparts. At least qualitatively, the presence of the chiral dopant has no significant effect on the sign of the surface anchoring. However, there were problems in using the DFPC coated droplets in switching studies. Although the droplets coated with 1 : 1 DOPC : DOPG were stable, after a few days, the MFPC and DFPC droplets began to coalesce and imaging using fluorescence microscopy showed that fluorescent ‘pockets’ had developed in the layer around each droplet. Because of these issues of stability for the DFPC coated 5CB-based droplets, attention was switched to an alternative method of controlling the surface alignment: control through the choice of the LC rather than through the choice of the phospholipid. Once again it was found that the results for the chiral-doped droplets paralleled those for their non-chiral counterparts.^22^ Hence, droplets of MLC7023 doped with S1011 or R5011 and coated with 1 : 1 DOPC : DOPG showed planar anchoring and a Frank–Pryce director field. These droplets were much more stable and practicable to use in studies of switching of the director field. Overall, the results of these studies are summarised in Table 1. These show the same alignment as previously reported for achiral systems by Paterson *et al.*^22^ and that the effect of changing either the lipid or the LC can be explained in the same way.

**Table 1:** Director fields observed for LC droplets formed from different LC materials, coated with phospholipid mixtures (all observations are at room temperature)

Material	PVA	SDS	DOPC : DOPG	DnPdPC	MFPC	DFPC
E7	Planar^a^	—	Perpendicular^b^	Perpendicular^b^	Perpendicular^b^	Perpendicular^b^
5CB	Planar^a^	—	Perpendicular^a^^,^^b^	Perpendicular^a^^,^^b^	Perpendicular^a^^,^^c^	Planar^a^^,^^b^
6CB	Planar^a^	—	Perpendicular^b^	Perpendicular^b^	Perpendicular^b^	Planar^b^
7CB	Planar^a^	—	—	—	—	—
MLC7023	Planar^a^	Perpendicular^a^	Planar^a^^,^^b^	Planar^b^	Planar^b^	Planar^b^

a Chirally doped LC droplets from this piece of work.

b Undroped droplets from previous studies.

c Predominantly perpendicular aligned droplets from previous studies.^22^

### Analyte switching of the director field in chiral-doped LC droplets

In the studies of the switching of the surface anchoring and of the director fields in chiral doped droplets; MLC7023 doped with 0.53 wt% S1011 coated with 1 : 1 DOPC : DOPG was used. This gives a long enough pitch to be directly observed using transmission mode microscopy and so it enables the evolution of the director field during the switching process to be observed in a direct manner. However, for the studies of the effect of switching on the optical properties of the system, using the chiral dopant S1011 at the much higher concentrations needed to give reflection of visible light was not possible because of the solubility limit of S1011 in MLC7023. Hence, for these studies, attention was switched to R5011, a chiral dopant with a higher helical twisting power, where lower concentrations are required and for which solubility in MLC7023 is not an issue.

**Fig. 11:**
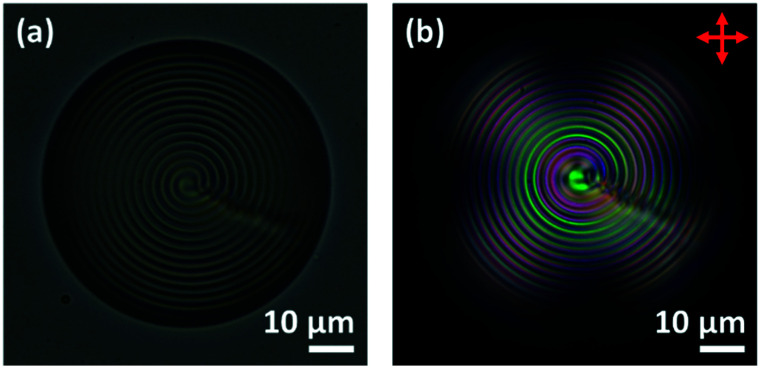
DOPC : DOPG coated droplets of MLC7023 doped with 0.53 wt% S1011 produced *via* a shaker method. (a) Transmission-mode image without crossed polarizers, the Frank–Pryce director field with concentric rings and a *s* = 2 defect line are clearly visible; (b) transmission-mode image with crossed polarizers.

**Fig. 12:**
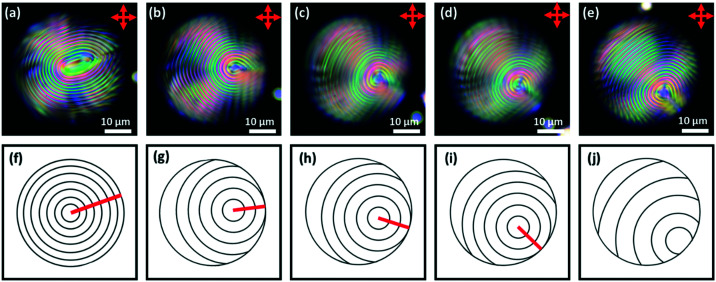
Top row (a)–(e) DOPC : DOPG coated droplets of MLC7023 doped with 0.53 wt% S1011 produced *via* a shaker method. 0.1 wt% SDS is added to the system and a transition is observed where the *s* = 2 defect shortens and the centre “spot” moves to the outer edge of the droplet. Each figure is taken at a 5 min time interval from when the SDS is added (image (a) 5 min, (b) 10 min *etc.*); bottom row (f)–(j) show schematic representations of the director alignment for the corresponding images above where one can observe the *s* = 2 disclination line shrink. *Some deviation from spherical isoclines was evident, for example in the elliptical nested cup of (a). This is likely due to the non-equilibrium nature of the photographs, taken as the switching process occurs.

Fig. 11 shows typical images of Frank–Pryce droplets of MLC7023 doped with 0.53% S1011 coated with 1 : 1 DOPC : DOPG having planar anchoring at the lipid interface. When the droplets are imaged by transmission-mode microscopy, and SDS is then added to replace the phospholipid and promote perpendicular anchoring, the director field evolves as shown in the top row of Fig. 12; it evolves through a series of ‘nested cup’ structures analogous to those shown in Fig. 1i, j, l and m. The switching processes shown as a series of still images in the top row of Fig. 12 can be viewed as a video in the ESI,† SV1.

**Fig. 13:**
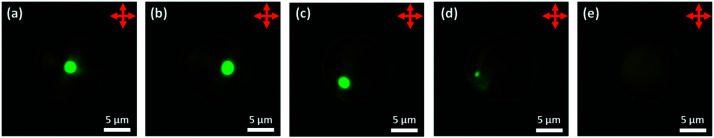
(a)–(e) Microfluidically produced DOPC : DOPG coated droplets of MLC7023 doped with 1.9 wt% R5011. 0.1 wt% SDS is added to the system and a transition is observed where the centre green “bullseye” moves from the centre of the droplet towards the edge before disappearing from the field of view. Each figure is taken at a 5 min time interval from when the SDS is added (*i.e.* the first images, (a) and (e), were taken after 5 min; when the transformation process had just begun).

**Fig. 14:**
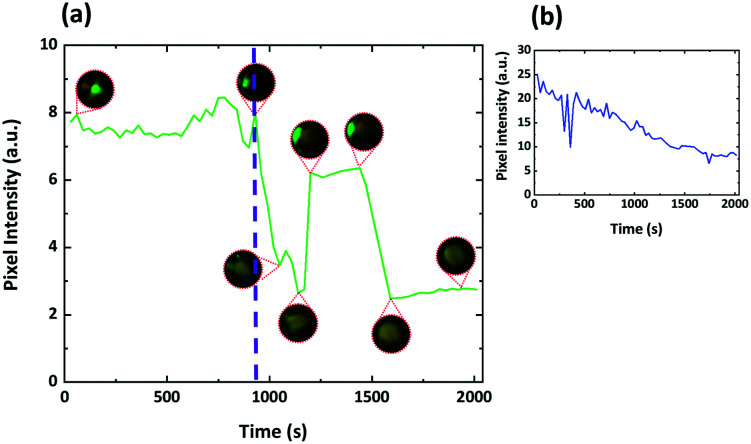
(a) Green pixel intensity data for the series of droplet stills in Fig. 13 and SV2.† Insets show actual droplet appearance between crossed polarizers at the indicated time stamps. The purple dashed line indicates the point at which the droplet alignment changes from planar to perpendicular. It is clear the tumbling motion of the droplet in solution has a much greater effect once the alignment has switched. (b) Shows data averaged over 30 droplets. Not all droplets switch at the same time and the stepwise elements of the response begin to be averaged out leading to the response shown.

The experiment was repeated using MLC7023 doped with 1.9 wt% R5011 (439 nm) coated with 1 : 1 DOPC : DOPG so that the effect on the optical properties of droplets that have a helical pitch comparable to that of visible light could also be studied. As shown in Fig. 13a–e, when the droplets are imaged using reflectance mode spectroscopy, what is initially observed is the typical, symmetrical bullseye. After SDS is added, and as switching progresses, this reflecting spot moves away from the centre of the droplet. As shown in Fig. 14, before the SDS is added the intensity of the reflected green light from the droplet is essentially constant; (other than the disclination line) the structure is symmetrical and its properties are essentially independant of its tumbling motion in the solution. However, after switching, the reflected light is found to vary strongly as the droplet tumbles in solution; it depends on the direction in which the ‘flashlight’ is pointing. For this system it was shown that the Frank–Pryce structure could be re-established, the structure could be switched back again by adding PVA and that it could be switched back again to a structure equivalent to that in Fig. 1j and m by adding a further amount of SDS; *i.e.* the process is fully reversible. The switching processes shown as a series of still images in Fig. 13 can also be viewed as a video in the ESI,† SV2.

**Fig. 15:**
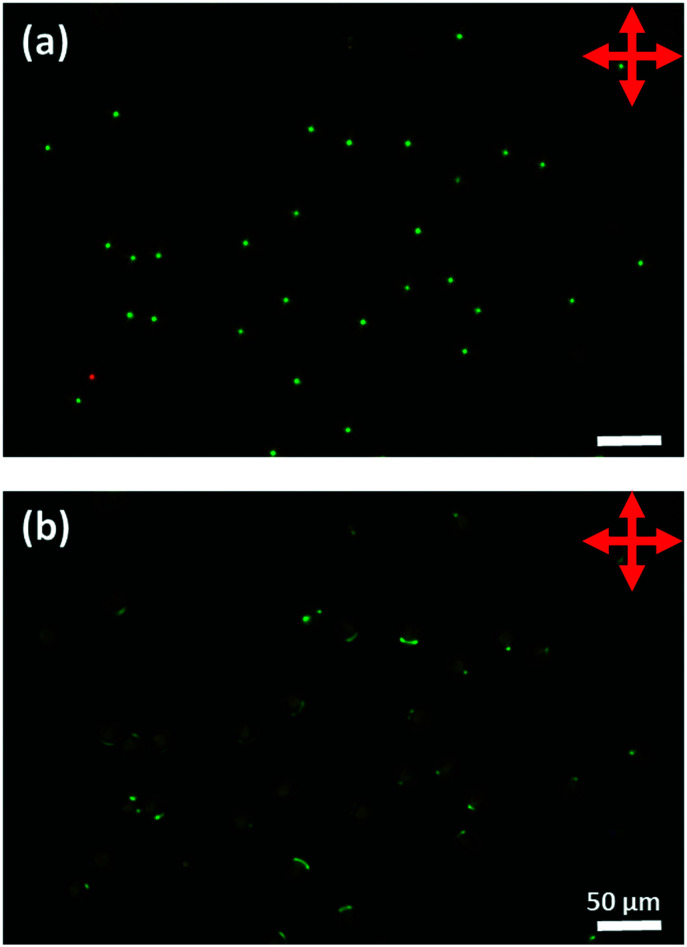
An array of microfluidically produced DOPC : DOPG coated droplets of MLC7023 doped with 1.9 wt% R5011. (a) Droplets in a Frank–Pryce alignment (LC aligned planar to the interface) with a clearly visible green reflection spot at the centre; (b) 30 min after 0.1 wt% SDS has been added and the alignment is no longer planar at the surface.

**Fig. 16:**
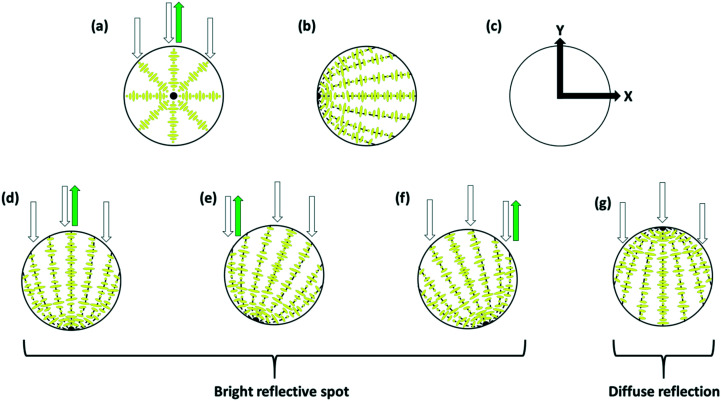
Schematic representation explaining the dependence of the reflected drop on the nature of the director field and the orientation of the droplet. (a) In the Frank–Pryce structure only in the centre of the droplet are the helical axes parallel to the incident light resulting in a bright reflective region in the centre of the droplet the position of which is insensitive to tumbling motion; (b) and (c) however the disposition of the chiral axes in the nested cup case is axially symmetric (symmetric about the *x* axis); (d)–(f) rotation/tumbling motions about the *z* axis result in the reflective spot moving left/right; (g) results in situations in which the only regions where the axis are parallel to the incident light are on the far side of the droplet giving a diffuse reflection (because of scattering of the reflected light by the intermediate layers). Rotations/tumbling motions about the *y* axis can result in movement of the position of the reflective spot up and down.

Because, after switching to perpendicular anchoring, the optical properties depend on the rotational state of the droplet, and because no sensor would be based on a single droplet, what is more relevant is the behaviour of a system containing many droplets averaged over time and space. Fig. 15 shows such results obtained by switching a number of droplets of MLC7023 doped with 1.9 wt% R5011 and coated with 1 : 1 DOPC : DOPG starting with planar anchoring and a Frank–Pryce/bullseye structure, and ending with structures with ‘perpendicular’ anchoring, Fig. 14. A video showing this switching process can also be viewed in the ESI,† video SV3. Note that, whereas for a single droplet the switching occurs over a relatively short timeframe, within an array of droplets they do not all switch at the same time. As a result the change in the optical properties is more continuous in nature.^30^ This is shown in the inset in Fig. 14. The period over which this change occurs will be dependant on the concentration of analyte and the kinetics of this type of response has been characterised previously.^20^ There may be some change in the wavelength or spread of wavelengths of the reflected light but, much more marked is the change in intensity. This can be understood in terms of the nested cup structure. As shown in Fig. 13, 14 and 16, for the Frank–Pryce structure reflection is independent of tumbling motions of the droplets in solution. The bullseye arises because only reflections from helical axes (almost) parallel to the incident light are observed. However, for the nested cup structures there is only strong reflection when the defect is on the opposite side of the droplet to the observer and in most cases this results in an off-centre reflective spot, Fig. 16e and f; since this is where the helical axes are roughly parallel to the incident beam. When the defect is on the same side as the observer, both the light entering the droplet and the reflected component (the only component involving helical axes parallel to the incident beam) must pass through the defect itself; through a region which is strongly scattering and there is only a weak, diffuse reflection. Hence, for an individual droplet, as it tumbles in solution the intensity of the reflected light shows step-like behaviour, Fig. 14; the flashlight goes on and off as the defect moves from the back to the front of the droplet. If switching were to an equatorial director field it is expected, on the basis of this argument, that there would be very low diffuse reflectivity.

When observing many droplets they do not all switch at the same time. The result is a sigmoidal dependence of the optical response with time, the form of which is dependant on the concentration of the analyte.^20^ However, with a sufficiently large number of droplets the fluctuations due to tumbling motions disappears. As shown in the inset in Fig. 14, even for just 30 droplets most of the fluctuations are lost.

## Conclusion

It is important to be able to control the surface anchoring properties, particularly for the phospholipid-coated LC droplets (droplets of the type relevant to the creation of biosensors). This is because, for most LC/phospholipid combinations, the phospholipid monolayer promotes such strong perpendicular anchoring that switching of the anchoring to planar, and the subsequent reorientation of the director field, is rather difficult. We have previously shown that judicious choice of either the LC or of the phospholipid enables this usual tendency towards strong perpendicular anchoring to be overcome. Here, the effect of these two components on the sign of the alignment is shown not to be noticeably affected by the presence of a chiral dopant and the results obtained for these chiral droplets follow those we previously obtained for their non-chiral counterparts. However, with chiral doped LC droplets, the ability to switch between highly reflective Frank–Pryce structures with planar anchoring and lower reflective structures with perpendicular anchoring suggests that they will prove much more useful in creating practicable sensor systems due to the strong optical signature associated with the change in director field. For single Frank–Pryce droplets with planar surface anchoring the intensity of the reflected light is essentially independent of the tumbling motion of the droplet in solution. However, when the surface anchoring is switched to perpendicular the intensity of the reflected light becomes dependant on the orientation. Although these effects have not yet been optimised, they suggest that chiral droplets could be used to create LC-based sensors analogous to LC thermometers; devices that are relatively cheap and disposable. In this respect, ongoing work has demonstrated that such droplets can be immobilized in films of hydrogel and further details of these immobilised systems will be published later. In more concentrated films than those described in this paper, in films where (it is hoped that) changes could be detected by the human eye, the effect of photonic crosstalk will become a much more significant factor. This component of the reflected light depends not only on the concentration of the droplets but also on the nature of their ordering.

## Author contributions

Experimental work was performed by D. A. P., X. D., P. B., and A. A. P. Analysis and interpretation was conducted by D. A. P, R. J. B., J. C. J., and H. F. G. The overall project coordinators are R. J. B., S. D. E., and H. F. G. All authors contributed to the writing of the manuscript.

## Conflicts of interest

There are no conflicts to declare.

## Supplementary Material

ME-007-D1ME00189B-s001

ME-007-D1ME00189B-s002

ME-007-D1ME00189B-s003

ME-007-D1ME00189B-s004
