# Supplementary material for: Chiral nematic liquid crystal droplets as a basis for sensor systems
Source: Mol Syst Des Eng. 2022 Mar 7;7(6):607–21. doi: 10.1039/d1me00189b (PMC9972830; doi:10.1039/d1me00189b)
Supplement: ME-007-D1ME00189B-s001 [file ME-007-D1ME00189B-s001.pdf]

Electronic Supplementary Material (ESI) for Molecular Systems Design & Engineering.
This journal is © The Royal Society of Chemistry 2022

Supplementary Information

Chiral Nematic Liquid Crystal Droplets as a Basis for Sensor Systems

Daniel A. Paterson,*[a,b] Xiaoxue Du,[a,d] Peng Bao,[a] Adele A. Parry,[a] Sally A. Peyman,[a,c] Jonathan A. T Sandoe,[c] Stephen D. Evans,[a] Dan Luo,[d] Richard J. Bushby,[b] J. Cliff Jones,[a] Helen F. Gleeson*[a]

[a] *School of Physics and Astronomy, University of Leeds, Leeds, LS2 9JT, UK*

[b] *School of Chemistry, University of Leeds, Leeds, LS2 9JT, UK*

[c] *Leeds Institute of Medical Research, University of Leeds, Leeds, LS2 9JT, UK*

[d] *Department of Electrical and Electronic Engineering, Southern University of Science and Technology, Shenzhen, 518055, China*

Contents

1. Energy comparison for rotating a LC droplet or reorienting its director field

Table ST1.

Table ST2.

Table ST3.

Figure S1. Plot demonstrating the cross-over point at which it becomes more energetically favourable to reorient the director rather than rotate the droplet.

2. Tables

Table ST4. Chiral LC mixtures.

3. Figures

Figure S2. Plots of the reflection spectra of 5CB doped with 7.3 wt% S1011 as a function of temperature.

Figure S3. Plots of the reflection spectra of E7 doped with 8.2 wt% S1011 as a function of temperature.

4. Videos

Video SV1. A video of a DOPC:DOPG coated droplet of MLC7023 doped with 0.53 wt% S1011 and switched with 0.1 wt% SDS.

Video SV2. A video of a DOPC:DOPG coated droplet of MLC7023 doped with 1.9 wt% R5011 and switched with 0.1 wt% SDS.

Video SV3. A video of an array of DOPC:DOPG coated droplets of MLC7023 doped with 1.9 wt% R5011 and switched with 0.1 wt% SDS.

1. Energy comparison for rotating a LC droplet or reorienting its director field

If we assume a 10 µm diameter droplet rotates at 2πs^{-1}:

The rotational kinetic energy of the droplet is given by;

$$U_r = \frac{1}{5} m r^2 \omega^2 \quad , \qquad (1)$$

Mass of droplet m;

$$m = \frac{4\pi}{3} \rho r^3 \quad , \qquad (2)$$

Hence;

$$U_r = \frac{4\pi}{15} \rho r^5 \omega^2 \quad , \qquad (3)$$

The strong r^5 dependence on droplet size is because the mass of the droplet increases with r^3 and the rotational energy increases with r^2.

Table ST1. Example values of rotational kinetic energy (U_r) for a droplet of 5CB (ρ = 1.01 kgm^{-3}) with droplet diameters of 10 and 20 µm.

**4π/15**	**ρ (kgm^{-3})**	**r (m)**	**ω (rads^{-1})**	**U_r (J)**
0.838	1.01 x 10^{3}	1.00 x 10^{-5}	6.28	3.31 x 10^{-21}
0.838	1.01 x 10^{3}	2.00 x 10^{-5}	6.28	1.06 x 10^{-19}

For a droplet of *r* = 10 µm, 3.31 x 10^{-21} J is needed to rotate the droplet once per second; whereas for a droplet of *r* = 20 µm, 1.06 x 10^{-19} J is need to rotate the droplet once a second. This is double the order of magnitude.

Reorienting the director field of a droplet

Approximating the droplet to a cylinder of dimension 2*r*, we can then approximate this to a circular section with a cell gap of 2*r*. Estimating that the director switches the bulk of the droplet in ~ 2V and that the change in capacitance is ~ 50% of Δε (which is ≈ 20);

$$U_n = \frac{1}{2}\Delta C V^2, \quad (4)$$

$$\Delta C = \frac{\pi r^2}{2r}\varepsilon_0 \Delta\varepsilon = \frac{\pi\varepsilon_0}{2} r \Delta\varepsilon, \quad (5)$$

$$U_n = \frac{\pi\varepsilon_0}{4} r \Delta\varepsilon V^2, \quad (6)$$

As the droplet increases in radius, so does the volume of fluid in which the elastic deformation is taking place. This will follow r^3. However, as r increases, the same elastic energy decreases and this will follow r^2.

Table ST2. Example values of director reorientation energy (U_n) for a droplet of 5CB with droplet diameters of 10 and 20 µm.

$\pi\varepsilon_0/4$	K (pN)	Δε	V (V)	r (m)	U_n (J)
6.91×10^{-12}	30	20	2.00	1.00×10^{-5}	5.53×10^{-15}
6.91×10^{-12}	30	20	2.00	2.00×10^{-5}	1.11×10^{-14}

Therefore, for a small droplet, it is easier to rotate the whole droplet than to reorient the director field.

*These values assume K ≈ 30 pN. A K ≈ 15 pN (closer to a real value for 5CB) is used below but the order of magnitude for U_n remains unchanged.

Cross-over droplet size

The point at which $U_n = U_r$ allows for a cross-over radius to be found;

$$r^4 = \frac{15\varepsilon_0 \Delta\varepsilon V^2}{16 \ \rho\omega^2}, \quad (7)$$

Table ST3. Example values of rotational kinetic energy (U_r) and director reorientation energy (U_n) for a droplet of 5CB.

4π/15	ρ (kgm^{-3})	r (m)	ω ($rads^{-1}$)	U_r (J)	$\pi\varepsilon_0/4$	K (pN)	Δε	V (V)	U_n (J)
0.838	1.01×10^3	1.00×10^{-5}	6.28	3.31×10^{-21}	6.91×10^{-12}	15	10	2.00	2.76×10^{-15}
0.838	1.01×10^3	2.00×10^{-5}	6.28	1.06×10^{-19}	6.91×10^{-12}	15	10	2.00	5.53×10^{-15}
0.838	1.01×10^3	4.00×10^{-5}	6.28	3.39×10^{-18}	6.91×10^{-12}	15	10	2.00	1.11×10^{-14}
0.838	1.01 x	8.00 x	6.28	1.08	6.91 x	15	10	2.00	2.21

	10^{3}	10^{-5}		x 10^{-16}	10^{-12}				x 10^{-14}
0.838	1.01 x 10^{3}	1.60 x 10^{-4}	6.28	3.47 x 10^{-15}	6.91 x 10^{-12}	15	10	2.00	4.42 x 10^{-14}
0.838	1.01 x 10^{3}	3.20 x 10^{-4}	6.28	1.11 x 10^{-13}	6.91 x 10^{-12}	15	10	2.00	8.85 x 10^{-14}
0.838	1.01 x 10^{3}	6.40 x 10^{-4}	6.28	3.55 x 10^{-12}	6.91 x 10^{-12}	15	10	2.00	1.77 x 10^{-13}
0.838	1.01 x 10^{3}	1.28 x 10^{-3}	6.28	1.14 x 10^{-10}	6.91 x 10^{-12}	15	10	2.00	3.54 x 10^{-13}

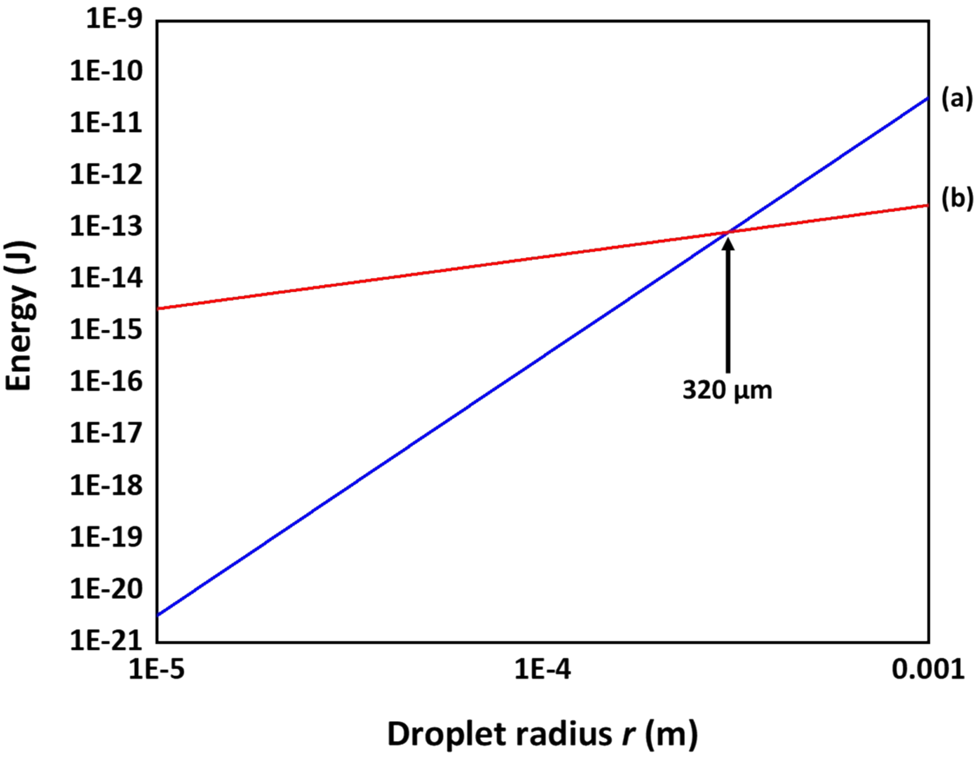

Figure S1. Plot of the cross-over point where it becomes more energetically favourable to reorient the director rather than the rotate the droplet. (a) Kinetic energy for rotation of a droplets of various sizes (U_r), (b) director reorientation energy for droplets of varying size (U_n).

It is clear that for droplets in the range 10 – 100 µm it will always be less energetically costly to rotate the whole droplet than to reorient the director field. Whilst these calculations have been done with an assumption that K ≈ 15 pN it is highly likely a similar trend would emerge for less elastically costly materials.

2. Tables

Table ST4. The compositions of samples and their calculated pitch values.

LC	S1011 concentration (wt%)	R5011 concentration (wt%)	Calculated pitch (nm)
5CB	7.3		343
6CB	7.3		343
7CB	7.4		337
5CB	0.53		4717
E7	8.2		305
MLC7023		1.9	439
MLC7023	0.53		4717

3. Figures

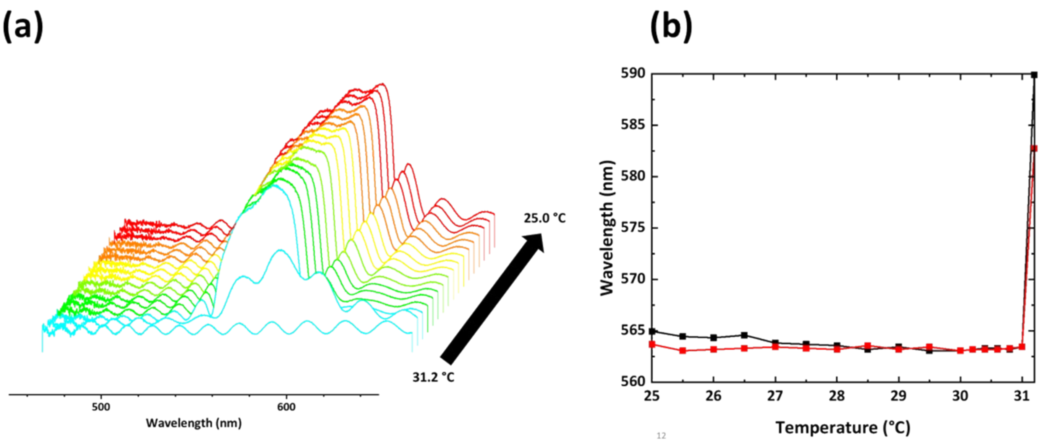

Figure S2. Temperature dependent reflection spectra of 5CB doped with 7.3 wt% S1011. (a) Reflection spectra obtained as the sample is cooled from the isotropic to 25 °C. The peak wavelength

is 544 nm, and the full width at half maximum is 55 nm. (b) The peak wavelength plotted against temperature showing no temperature-dependence.

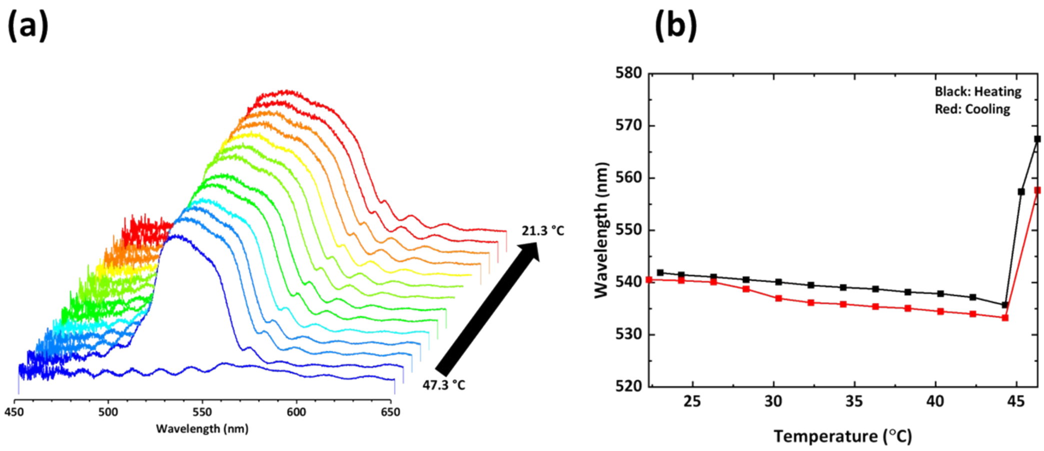

Figure S3. Temperature dependent reflection spectra of E7 doped with 8.2 wt% S1011. (a) Reflection spectra obtained as the sample is cooled from the isotropic to 21 °C. (b) The peak wavelength plotted against temperature showing no temperature-dependence.

4. Videos

Video SV1. A video of a DOPC:DOPG coated droplet of MLC7023 doped with 0.53 wt% S1011 and switched with 0.1 wt% SDS.

Video SV2. A video of a DOPC:DOPG coated droplet of MLC7023 doped with 1.9 wt% R5011 and switched with 0.1 wt% SDS.

Video SV3. A video of an array of DOPC:DOPG coated droplets of MLC7023 doped with 1.9 wt% R5011 and switched with 0.1 wt% SDS.
